# Pathology and Treatment of Psoriasis Using Nanoformulations

**DOI:** 10.3390/biomedicines11061589

**Published:** 2023-05-30

**Authors:** Divya Thirumal, Rakesh K. Sindhu, Shuchi Goyal, Aayush Sehgal, Ashok Kumar, Marianesan Arockia Babu, Pradeep Kumar

**Affiliations:** 1 Chitkara College of Pharmacy, Chitkara University, Rajpura 140401, Punjab, India; 2 School of Pharmacy, Sharda University, Greater Noida 201310, Uttar Pradesh, India; 3 Department of Pharmacology, G.H.G. Khalsa College of Pharmacy, Gurusar Sadhar, Ludhiana 141014, Punjab, India; 4 Department of Cardiology, Sadbhwana Hospital, Fatehabad 125050, Haryana, India; 5 Institute of Pharmaceutical Research, GLA University, Mathura 281406, Uttar Pradesh, India; 6 Wits Advanced Drug Delivery Platform Research Unit, Department of Pharmacy and Pharmacology, Faculty of Health Sciences, School of Therapeutic Sciences, University of the Witwatersrand, Johannesburg 2193, South Africa

**Keywords:** psoriasis, bioactive, drug delivery system, nano formulations, therapies

## Abstract

Psoriasis (PSO) is an inflammatory skin condition that causes a variety of diseases and significantly decreases the life characteristics of patients, and substantially diminishes patients’ quality of life. PSO usually impairs the skin and is linked to various disorders. Inflammation pathology does not only damage psoriatic skin; it shows how PSO impinges other body parts. Many variables interact with one another and can impact the etiology of psoriasis directly or indirectly. PSO has an effect on approximately 2% of the world’s population, and significant progress has been made in comprehending and treating the alternative PSO by novel drug delivery systems. Topical, systemic, biological, biomaterials, and phototherapy are some of the useful therapies for PSO. Nonetheless, topical treatments remain the gold standard for treating moderate PSO. The applicability of several nanocarrier systems, such as lipid nanoparticles, metallic nanoparticles, and certain phytocompounds, has been briefly explored. The present review focuses mainly on traditional therapeutic strategies as well as on breakthroughs in nanoformulations and drug delivery methods for several anti-psoriatic drugs.

## 1. Introduction

**Figure 1:**
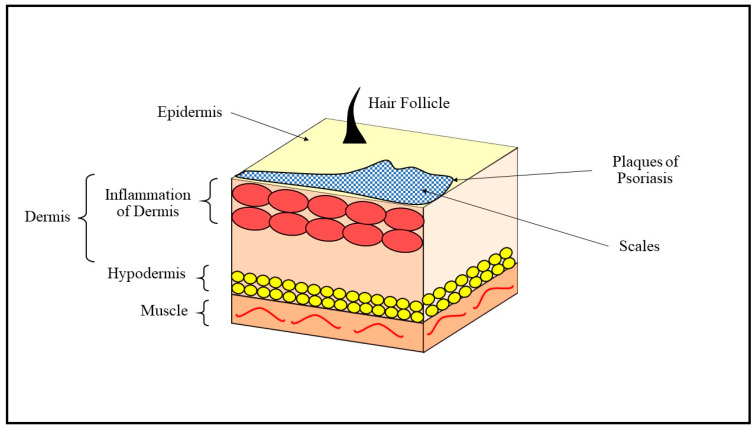
Chronic inflammation leads to the formation of characteristic plaques on the dermis, illustrating the pathology and pathogenesis of the dermis and hypodermis, leading to the development of plaques and scales on the epidermis, with underlying muscles impacted.

PSO is an immune-mediated disease with a genetic and environmental basis where the stimulation and inflammatory responses produce the proliferation and differentiation of keratinocytes [1]. PSO affects about two percent of the entire global population, and significant progress has been made in understanding and therapeutic approaches to PSO. Just 19% of countries possess data on the epidemiology of PSO. The occurrence of psoriasis among youngsters is less than one percent in every country, and it varies by geographic region. Norway has the highest prevalence of psoriasis, accounting for 1.98% of total inhabitants, while the Western population has an overall incidence of around 2%, with the lowest frequency of 0.12% in East Asia [2]. Consequently, PSO can be defined as an epithelial disorder characterized by red scaly patches that most often appear on the elbows, knees, scalp, and lower back, though any area of the skin can be affected, as shown in Figure 1. There is also complexity, including psoriatic arthritis, cardio-metabolic disorders, and psychological disorders [3]. The importance of targeting keratinocytes for PSO therapy systems has a crucial role in beginning and sustaining the inflammatory state, including epigenetic alterations. PSO can be distinguished by the activation of human keratinocyte (HaCaT), which multiplies up to 10 times faster than normal epithelial cells. Cells with antigens, the primary dendritic cells, are triggered by the distal antigens produced by HaCaT in response to injury. Keratinocyte cells use pattern recognition receptors to detect external or pathogenic contaminants, also tissue damage. The activation of HaCaT leads to the generation of secondary intermediates like inflammatory cytokines and bacteriocins. Antioxidant, antispasmodic, antiproliferative, and antibacterial properties are some characteristics of intrinsically therapeutic NPs (ITNPs), which are used medicinally to cure a variety of illnesses. Pharmacological medications such as biologics or natural compounds are new therapy techniques that have proven to be more effective than traditional therapies for mild to serious PSO [4]. Since unprecedented approval was given to etanercept by the Food and Drug Administration (FDA) in 2004, biological agents altered the new PSO therapy paradigm [5]. Nanoparticles (NPs) are intriguing, novel, innovative nanoscale methods used for improving active pharmaceutical ingredients (API) distribution to the specified site. The studies reveal that diverse APIs can be enclosed into various NPs. However, liposomes and nano-capsules are the most efficient nano-based pharmaceuticals designed for delivering PSO therapies. Anti-psoriatic drugs increase skin permeability and reduce PSO sensations when combined with lipid-based nanostructures. Furthermore, the treatments demonstrate significantly favorable results in treating PSO sores due to their enormous porous structure, which improves percutaneous absorption, persistence, and extended distribution. Solid lipid nanoparticles (SLNPs) and lipoprotein nanostructures (NLCs) are colloidal carriers that help with bioactive component dosage and distribution. SLNPs and NLCs are excellent choices for specific target medication delivery. Approximately 80 percent of affected individuals utilize some type of conventional therapy, which typically struggles from poor medication diffusion, which is additionally limited by hyperpigmentation and a loss of hydration in the lesional skin [6]. Since these therapies persist at the PSO region for an extended period, nanomaterial compositions should maintain the NPs on the afflicted area throughout the API discharge, the most recent strategy for superficial psoriatic therapy. Keratin peptides are the most utilized hydrocolloids as surgical instruments and external drug carriers because they are not only a biocompatibility element of the human extracellular matrix (ECM), but they also proactively assist in the injury healing pathway [7,8]. PSO, notably the advanced stage, is strongly linked to cardiovascular disease, metabolic syndrome, and inflammatory illnesses. PSO prevalence varies from modest, with a small number of discrete inflammatory melanomas, to severe, with extensive patches covering over 10 percent of the body surface area [9]. Topical medications are employed temporarily to relieve moderate PSO and to manage intermediate to chronic PSO [10]. The dermal, lingual PSO drugs, as well as innovative PSO treatments and siRNAs, address the genetic variations in PSO etiology. Several investigations have shown that nanocomposites improve efficiency and lessen the negative impacts of the medications they carry by increasing epidermal preservation, sustaining ejection, and reducing systemic penetration. Chemokines implicated in the ailment are raised in PSO clinical signs, and the complexity of the condition can relate to a number of mediators in serum and neovascularization within the dermis. Some studies show that different APIs can be integrated into different lipid nanostructures, but liposomes and nano-emulsions have the most effective micro-drugs formulated for PSO treatments. Anti-psoriatics associated with SLNPs increase skin permeability and reduce the symptoms of PSO. In addition, treatments show significantly favorable results in the treatment of PSO lesions due to their huge nanoscale surface area, which enhances skin permeability, persistence, and long-action effects. However, further studies and possibilities with other forms of SLNPs, such as niosomes, transfersomes, and ethosomes, are explored for their immense potential in topical anti-psoriatic administration. The creation of nanomedicines is also linked to cytotoxicity and safety problems but can be solved through a thorough understanding of how they interact in the human body [11]. The new formulations based on nanocarriers are a great promise to overcome the challenges faced by traditional formulations, which are shown by offering lower doses, dosing frequency, dose-related adverse effects, and increased efficiency of APIs. Nanoformulations are now widely used in the safe and effective treatment of PSO [12].

## 2. Pathology and Pathogenesis of Psoriasis

PSO is a severe autoimmune cellular T-mediated dermatitis caused by epithelial hypertrophy, abnormal epithelial stratification, and inflammation infiltrates, with a characteristic Th1 and Th17 being dominant [13]. PSO is proposed as a clinical syndrome rather than a skin disorder [14]. PSO has been widely regarded as an autoimmune disease in which a variety of lymphocytes and T cells play crucial roles. PSO causes the activation of innate immune cells and pathogenic T lymphocytes, resulting in skin conditions and HaCaT hyperproliferation. B cells have generally been neglected when it comes to their importance in the genesis of PSO. T cells play a critical role in the emergence of PSO; however, the method of activation of T cells cannot elucidate all the characteristics of the disease. Various mediators, as well as the biological involvement of other cells, such as neutrophils, macrophages, keratinocytes, and B cells, contribute to an increasingly complicated chain of occurrences that eventually results in the creation of PSO [15]. Platelet linkages with lymphocytes are documented in psoriatic experimental animals and are common with numerous other inflamed illnesses. No function for platelets in PSO individuals’ lesions has been identified [16]. However, a significant correlation between platelet and monocyte aggregation in blood circulation and skin diseases is consistent with the systemic inflammatory nature of PSO. The regulation of pro-inflammatory cytokines, including tumor necrosis factor (TNF-1), interleukin-1 (IL-1), and class I interferons (IFN), is related to PSO lesions [17]. The immune–competent inflammatory lesions skin model showed a gradual onset of epithelial distinction, hyperproliferation of basal keratinocytes, an increase in pro-inflammatory cytokine secretion, and a disruption in the expression of important transcriptional regulators, as seen in lesional plaques, indicating the crucial significance of incorporating the trait of cutaneous molecules to T cells to produce a pertinent prototype for PSO [18]. The defective innate and adaptive immunological mechanisms that drive PSO pathogenesis are also known to increase insulin sensitivity, atherosclerosis, and coagulation. PSO is classified under four types based on diagnostic characteristics: plaque PSO, erythrodermic PSO, guttate PSO, pustular PSO, etc. [19]. Psoriatic inflammation is caused and maintained by disruptions in the naturally occurring, adaptive epidermal immune systems. In certain cases, innate immune system activation is caused by innate surface receptors and cytokines coexisting with autoinflammatory perpetuation, whereas in others, T cell-driven autoimmune responses occur. Hence, PSO exhibits autoimmune disease characteristics on an inflammatory foundation, with both pathways overlapping and even amplifying one another [13]. The revelation of T cells in the cause of PSO shifted back to keratinocytes, whereas the discovery that patients with genetic variations in some HaCaT genes or mice with particular effectiveness of some HaCaT genes spontaneously develop PSO shifted the back to keratinocytes. Regardless of the cause of PSO, the interaction between HaCaT and lymphocytes is essential in the etiology of the disorder [20]. The identification of the involvement of interleukin-17 (IL-17) and IL-23 in the progression of psoriatic illness has resulted in significant improvements in our comprehension of the pathogenic immunological processes in PSO, as well as a paradigm change in the management of this illness [21]. Types of PSO:

- Plaque PSO:
  − The most prominent type of the condition.
  − This mainly affects 70–85% of people.
  − It commonly appears on the wrists, knees, scalp [22], and lower spine.
  − CAUSES: Dermal scraping, infections, medications, alcoholism, psychological anxiety, smoking, and exposure to radiation [23].
- Flexural PSO:
  − Also recognized as inverse PSO.
  − It can be observed in the armpits, groin, breasts, and various skin crevices surrounding the genitals and buttocks.
  − PSO affects approximately 20% of the population.
  − CAUSE: Yeast proliferation, extreme hypersensitivity to abrasion or perspiration [24].
- Guttate PSO:
  − Typically provoked by a microbial infestation.
  − Microscopic drop lesions appear on the torso, limbs and scalp.
  − CAUSE: Streptococcal infection, microbial infections, skin injuries, such as cuts, blisters, or insect bites, medications, Sun damage, psychological tension, and alcohol intake [25].
- Nail PSO:
  − Irregular nail development and discoloration can occur in fingernails and toenails. Color changes, small pits, lines across the nails, a white area on the plate, thickening of the skin under, and loosening of the nails are all symptoms.
  − CAUSE: A confluence of inherited, ecological, and immunological factors [26].
- Psoriatic arthritis:
  − An inflammatory condition that affects the joints of children and adults with PSO.
  − Red, swollen, tender, warm, and stiff joints, stroke, atherosclerosis, myocardial infarction.
  − CAUSE: Trauma or injury on the skin, like cuts or burns, medicines, alcohol, skin irritants, and smoking [27].
- Erythrodermic PSO:
  − Defined by recurring, intense erythema of the epidermis and the accumulation of scales in layers instead of tiny flakes.
  − Increased cardiac pace, changing body temperature, skin reddening.
  − CAUSE: Corticosteroid usage, burns, psychological pain, drinking, illnesses, and allergies [28].
- Pustular PSO:
  − Dermal reddening, accompanied by pustule development and scaling. Discomfort or light sensitivities.
  − CAUSE: Excessive UV radiation, pregnancies, steroids, diseases, extreme trauma, and abrupt discontinuation of systemic medicines or powerful external stimulants [29].

## 3. Mechanism of Percutaneous Absorption and Treatment

**Figure 2:**
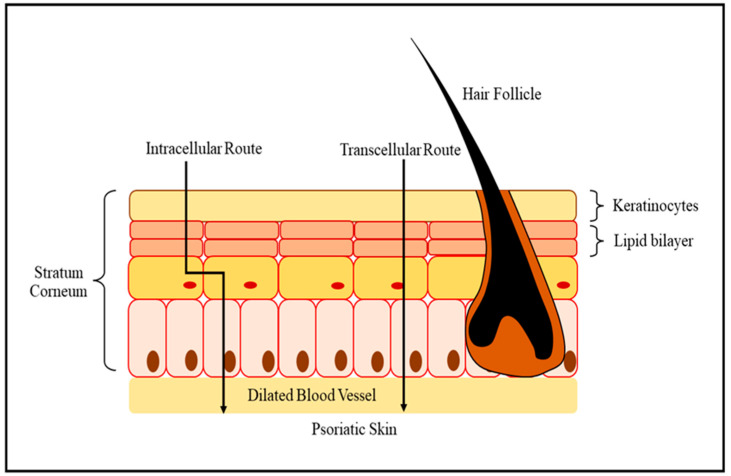
Pathways implicated in the mechanism of psoriasis: understanding the intracellular and paracellular routes involved in the abnormal differentiation and activation of keratinocytes, formation of dilated blood vessels in the stratum corneum.

The general basis for treating mild to severe PSO is topical therapy. It enables target treatment of the specific affected skin while preventing systemic side effects. However, patient satisfaction with available treatments remains low [30]. Cutaneous availability is a critical aspect of topical therapy. The qualities of the skin, the physicochemical characteristics of the medication and the carrier, and the interaction of the medication as well as its vehicle with layers of skin, all influence topical drug delivery. Hydrophilic compounds with a molecular mass of less than 500 Daltons often penetrate undamaged skin. This explains why molecules that are extremely hydrophilic or lipophilic, as well as molecules with higher molecular weight, are less suitable for treatment with traditional topical drugs [31]. The dermis is the primary layer of the skin, and its significance to skin metabolism is significant because of its varied cellular proliferation, vascular, and secretion of biochemical intermediaries implicated in extracellular matrices preservation and immunological reaction control. Trans epidermal and trans appendageal pathways can both be used for transdermal administration, as shown in Figure 2 [32]. The transcytosis pathway of a skin cell is made up of hydrophilic areas that are enclosed by triacylglycerols which form the sidewalls of nanopores. Substances passing via this channel enter corneocyte groupings using flaws that form water-filled holes. Most chemicals or particles enter the epidermis via intercellular diffusion within stratum corneum (SC) corneocytes. Because corneocytes are not arranged perpendicular to each other in strata, a chemical must transit through a tortuous course when permeating between them. This channel is hypothesized to allow free-volume transport across the phospholipid bilayer found between cells [33]. Topical application of resveratrol and oligomers was characterized in vivo by assessing cutaneous absorption, skin physiology, pro-inflammatory mediator expression, and histopathology in IMQ-treated mice. Skin deposition decreased as the molecular size and lipophilicity of the permeants increased. Resveratrol exhibited the highest absorption, followed by ε-viniferin. Skin delivery can be influenced by the lipophilicity, molecular size, and steric structure of chemicals. The oligomers of resveratrol provide ideal candidates to explore the impact of molecular size on skin permeation. Cutaneous absorption of permeants is strongly related to molecular structure. Cutaneous absorption depended on the steric structure and physicochemical features, as well as the vehicle. Skin deposition decreased as molecular size and lipophilicity increased; the monomers exhibited greater flux than the oligomers. Absorption of the oligomers, especially the tetramer was significantly increased in barrier-defective skin. Topical ε-viniferin more potently reduced hyperplasia and inflammation than resveratrol [34]. The different nano-sized bioactive treatments used in cosmeceutical therapy as depicted in Table 1.

## 4. Drug Delivery Systems for the Treatment of PSO

Microparticles, microemulsions, micelles, and microcapsules are explored as delivery mechanisms for PSO treatment. PSO is a dermatological condition that is becoming more prevalent around the world. The existing therapeutic options relying on traditional preparations are non-specific and linked with toxicities. A novel medication administration strategy based on nanoformulations (NFs) could provide an effective opportunity for the creation of reduced toxicity and large-element therapy strategies. It has easier access to the epidermis and allows for better absorption. Nanocomposites boost the surface volume proportion, culminating in improved penetration along all channels via the skin, particularly intra-cellular, inter-cellular, and trans-appendage routes. Nanospheres are being widely researched for use in directed medicine administration: for example, liposomes, NLCs, niosomes, SLNPs, ethosomes, transfersomes, nanosuspension, and dendrimers have also been described in the classification of PSO. Because of their membrane form, NFs can be fashioned into sticky anti-PSO treatments, whereas nanogels could offer a considerably moister habitat to the affected epidermis. Topically applied medication release using these technologies alone might be ineffective. Thus, researchers have paired NPs with NFs [39] and hydrogels for highly efficacious PSO therapies.

Microspheres are rigid spherical granules ranging in size from 1 to 1000 μm that comprise scattered medicinal ingredients in fluid or crystallized conditions. They are free-flowing, deep round particles made of disposable protein and synthetic polymeric materials [40]. Porous polyamide microspheres can be made to convey a variety of medications and disperse those over the epidermis. By employing empty microspheres, the thickness of the substance could be reduced. Reliability, drug distribution, and absorption can all be altered by the sort of material utilized [41].

Microemulsions are granular isotropic pharmaceutical methods composed of both oil and water, with surfactants and co-surfactants providing thermodynamic consistency. They vary in size between 20 to 200 nm, have a little opalescence, and are transparent in nature [42]. Such colloidal transporters increase the epidermal distribution of all lipophilic and hydrophilic chemicals. Drug localization and distribution in the epidermal strata are clearly demonstrated by microcapsules [43].

Micelles are aggregate colloid systems composed of amphiphiles that self-assemble to produce a unique fundamental configuration above the crucial micellar intensity. The size spectrum extends from 5 to 200 nm. Polymeric micelles are formed by the mixture of polar and non-polar copolymer and dimer groups in a liquid environment [44]. The hydrophilicity nature of the shell in the repository lines the liquid media in micelles, while the polar interior of the reserve aids in the solubilization of poorly soluble substances. Polymeric micelles can be employed in medication and genome administration methods via oral, ophthalmic, injectable, topical, and intranasal modes [45].

Microsponges are innovative therapeutic delivering devices composed of perforated microparticles. They are made up of hydrogel granules with a large surface area. This distribution method will improve therapeutic efficacy, eliminate undesired, unintended effects, and alter the drug’s clearance in the organism. The medication is released from the microsponges amongst the epidermal discharges. Its permeable nature allows the media to enter it. The medicine degrades and is delivered when the released medium permeates the microsponges. The substance first comes in contact with the microsponge interface, then travels into the interior region, allowing through the encapsulated medicament in the apertures, resulting in its prolonged emission [46].

Microneedles have the restricted penetration of medications via the epidermis represents the key disadvantage of the topical administration technology. A crucial unaddressed difficulty is gaining substantial medication absorption into the inner levels of the dermis. Several administration technologies that improve pharmaceutical transdermal release have lately been created [47]. Microneedles have the potential to distribute drugs transdermally, and this strategy can alleviate the major issues encountered with traditional topical administration technologies. The use of microneedles can increase medication penetration via the epidermis. This method can be used to supply a wide range of hydrophilic medicines. The microneedle gadget is made up of micron-sized needles that are organized on a patchwork. This needle technology will puncture the skin barrier, removing the impediment to medication penetration. Then, the medicine is incorporated directly into the skin, where it can readily enter the bloodstream [48].

NLCs are phospholipid transporters of the successor that include both fluid and firm lipids. The pharmaceutical carrying capability into the matrices is increased in this fashion by disrupting the polymer [49].

Liposomes are bilayer transporters made up of lipoprotein, regulators, and lipid membranes. Lipophilic and hydrophilic compounds can be carried by them. The diameter of particles can fluctuate based on their shape [50]. Following their administration, systemic absorption is reduced, and negative consequences are prevented. The location of liposomes in the stratum corneum increases medication agglomerates in the epidermis [51].

Niosomes are spherical shell amphoteric entities with a bilayer structure. These are classified as matrices, primitive cell models, and cell-like bioreactors for bio-encapsulation. In recent times, traditional lipid membranes have been transformed into niosomes that are thought to have the possibility for directed medication application [52].

**Figure 3:**
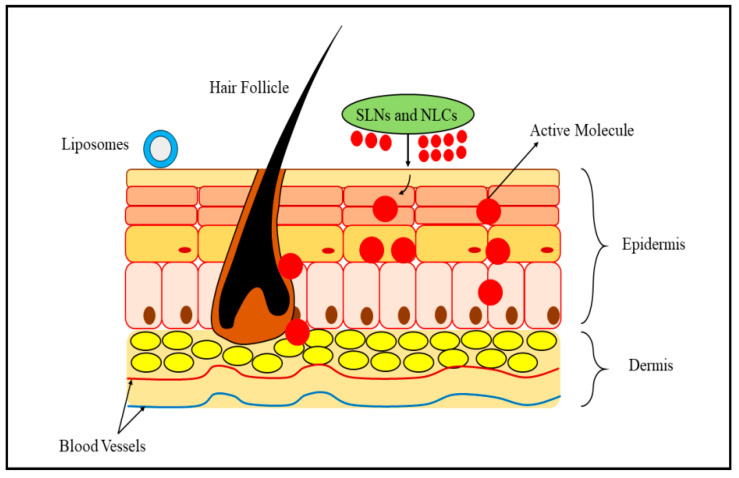
Mechanism emphasizing the impact of liposomes, SLNs and NLCs on the epidermal layer and dermal layers of psoriatic skin.

SLNPs are composed of liposomes and emulsifying agents that are distributed in an aqueous phase, and their standard area ranges in the nanometric scale [53]. They remain rigid at ambient temperatures [54]. This size variety improves drug penetration through the epidermis by supplying a broader area of contact and increased effectiveness as a medication distribution framework [55]. The benefits of SLNPs include improved physiological consistency, the capability to incorporate hydrophilic and lipophilic medicaments, a lower expense than liposomes, and the simplicity of production and its scale-up [56]. In the biopharmaceutical industry, SLNPs have been used to regulate the therapeutic release and increase the accessibility of preloaded active ingredients by modifying the dissolution pace in injectables such as intravenous, intramuscular, or hypodermically, edible, and vaginal therapies. They are used in optometry, dermatitis, and cosmetology [57]. The mechanism of liposomes, SLNs and NLCs on the epidermal layer and dermal layers of psoriatic skin is shown in Figure 3.

Ethosomes give a potential vesicle composition with a strong compressibility property. They can carry drugs deeper within the epidermis via the stratum corneum more efficiently than lipid nanoparticles [58]. The particulate diameter dispersion is irregular, with fragments ranging from 30 nm to microns remaining restricted to the epidermal [59]. The main benefits are enhanced medication penetration and good user adherence. Furthermore, the composition constituents are non-noxious, and medication distribution should not be complicated [60]. Ethosomes can improve the permeation of medications that are not easily absorbed via the surface. One common case is MTX, which is employed to alleviate psoriasis.

Transfersomes are made up of triglycerides and transmembrane-relaxing substances. The significant aspect of the wall is lipids, and the transfersomal layer is devastated by compounds known as edge activators. Liposomes and transfersomes have the equivalent morphology. Surprisingly, transfersomes are extremely versatile and can readily penetrate apertures by shrinking in diameter when contrasted to their initial dimensions. As a result, these can be classified as flexible lipid nanoparticles. Medications that are hydrophilic dissolve in the interior liquid environment, whilst medications that are lipophilic and amphiphilic become entangled in the bilayer barrier [61].

Dendrimers are 3-D macromolecule design categories containing diverse bifurcation sections, potentially multiple terminating domains, and an activator center [62]. These biomolecules can be changed together into a variety of potential designs for the aim. Formerly, biodegradable aptamers were investigated for the administration of anticancer medications such as doxorubicin and cisplatin [63]. These are also being used to administer anti-psoriatic medicines.

Ultimately, these clinical trials are investigated to assess the safety and efficacy of metallic NPs, calcipotriol-loaded NLCs, betamethasone valerate-loaded SLNs, and the usage of MTX-loaded liposomes in the management of PSO by lowering psoriatic manifestations. If productive, these NPs are transformed into PSO therapy by enhancing the distribution of APIs and decreasing adverse symptoms.

Skin design and intricacy impede the transit of chemicals, encouraging researchers to investigate various kinds of NPs and some phytocompounds which permeate the epidermis via various methods for ailment therapies and dermal-cosmetic uses:

### 4.1. Nanoparticles

Nanoparticles (NPs) are the key topic that has been widely researched as an efficacious carrier for therapeutics in nano-sized formulations. NPs have a high surface-to-volume proportion and often pass via the skin’s apertures in the hair shaft. Particularly, NPs transmit hydrophilic and hydrophobic compounds deeply into the epidermis via the lipid compounds pathway, transcytotic, or follicular site. The impact of medication penetration through NP transporters is determined by the diffusion rate, lipid-water partition coefficient, charge density, and pH of the NPs. NPs with a diminutive diameter, a low molecular weight, positive ions, and a neutral pH are shown to be more prone to reach the skin [64].

### 4.2. Lipid Nanoparticles

Researchers have produced many forms of lipid nanoparticles by studying the integrity biology of intercellular lipid membranes, including SLNPs, nanovesicle-carrying nanostructured lipids, and nano-capsules. Because of the bioavailability of the nano-sized molecules, these innovative drug delivery methods enable inadequately solubilized active medicinal ingredients to reach the targeted spot effortlessly. Due to the amphiphilic nature of lipids, which readily enclose both lipid-soluble and hydrophilic medicines, SLNPs for PSO therapies provide a paradigm for topical drug delivery.

Lipoprotein nanostructures have been proven to be excellent carrier systems capable of increasing tripterin absorption, which is an anticancer compound. NLCs are the most recent category of liposome nanostructures, and they have grown in popularity during the last decade. NLCs are made up of a combination of both liquid and solid lipids that dissolve the APIs and are maintained by a solvent. Solubility of lipid adjuvants and compositional alterations have crucial roles in formulating durability and are difficult to anticipate in the initial stages of pharmaceutical manufacturing. Though the components are morphologically soluble, micro variability throughout preservation will cause dissociation, which will be visible only after several months of stability observations [65].

### 4.3. Metallic Nanoparticles

Such new nanocarriers considerably increased the inhibition activity of MTX on epithelium in-vitro when contrasted to one MTX therapy. The research presented initial evidence for the use of AuNPs in transdermal medication administration. Investigators could use AuNPs to extensively examine the fundamental principles through which the medication produces its potential. AuNPs derivatized with 3-mercapto-1-propanesulfonate and preloaded with MTX were also manually injected in vivo and demonstrated curative results on psoriasiform in a separate investigation [40]. Crisan et al. revealed that AuNPs and AgNPs crosslinked with Cornus mas which is a polyphenol-rich extract that had anti-inflammatory properties in-vitro on bone mesenchyme murine macrophages as well as anti-psoriatic efficacy in individuals [66].

### 4.4. Phytocompounds

In contrast to the references hydrogel, the investigation results showed much higher acitretin accumulation in adult dead tissue from the acitretin-NLC gel. The scientific trial revealed a significant improvement in treatment efficacy as well as a diminution in acute toxicity with acitretin-NLC-gel. Curcumin (Cur), a natural polyphenol produced from turmeric, has lately attracted interest in skin-related illnesses for its anti-inflammatory effects. Nevertheless, direct Cur therapy is ineffective due to its hydrophobicity, volatility, and limited absorption. Furthermore, hyperkeratosis and a paucity of hydration in psoriatic skin can cause limited diffusion, which prevents abilities from penetrating the stratum corneum. For this reason, a polymer-based Cur composition was enabled for the external management of PSO. Cur was originally enclosed in chitosan NPs (CS-NPs) to increase its thermodynamic durability. The Cur-loaded nanoparticles were embedded in an aqueous, biodegradable collagen-based patch. CS-NPs are notable in cutaneous preparations [67,68,69,70,71]. CS is a nature-generated polymeric material with bioactivity, low immunogenicity, degradability, mucoadhesivity, in situ gelation, permeability intensification, and innate antibacterial potential [72]. It is commonly employed for transporting Cur either individually or in conjunction with other constituents. For instance, Cur was entrapped using a zwitterionic inclusion complex of ionized CS and negative charge acylated cruciferin. Cur-loaded CS-NPs of 167–251 nm demonstrated higher encapsulation efficacy, epidermal penetration, drug dissolution, and epigenetic changes in transdermal delivery [68]. The initial step is the production of Cur analogs with better physiochemical attributes and therapeutic actions, which includes curdiethyl disuccinate and cur diglutaric acid [48]. The other method is encapsulating in CS/Alg NPs, a sustainable medication that also boosts cur potency [73]. Reduced permeation and increased component volatility of diverse personal care treatments for prolonged and increased component administration to cosmetic health sciences are important challenges in the use of plant-based pharmaceuticals. In order to address this challenge, nanometric methods are presently being used in cosmetic industries and products for prolonged and increased administration of phytol-derived beneficial chemicals. Aloe vera, curcumin, resveratrol, quercetin, vitamins C and E, genistein, and green tea catechins have been extensively nano-sized and included in numerous gels, lotions, and creams for skin, lip, and hair maintenance for their long-term impacts. Flavonoids help to reduce skin irritation. The possibilities of treating PSO with topical application of flavanone and its metabolites, such as naringenin, hesperetin, 6-hydroxy flavanone, flavanone, and 6-bromo flavone to understand the relationship amongst structure and permeability, the physiochemical and structural properties of the substances were computed using molecular modeling. We obtained a result where flavanone had the highest skin penetration of the chemicals. Flavanone reduced IL-6 overexpression in psoriasiform lesions by 80%. Naringenin restored the skin condition and was measured by transepidermal water loss (TEWL). Tea catechins and associated chemicals can be delivered more efficiently via nanotechnology. High catechin load-carrying effectiveness, maintained or longer delivery, greater catechin integrity, enhanced bioavailability, and accelerated deposition or localization to the nidus are all benefits of catechin-loaded nanocarriers for external administration. Intimate skin interaction, enhanced skin moisture, skin structural disruption, and follicular absorption can all promote catechin penetration [74]. Various phytocompounds treatments used in treatment of various diseases as shown in Table 2.

## 5. Current Therapies

Dermal medicines are used to treat restricted or centrist distress PSO, whereas light or medical treatment is used to treat modest conditions [85]. Traditional psoriatic medication approaches may incorporate both the circulatory and cutaneous release of curative drugs. The previously used medicines are typically associated with low oral absorption due to hepatic first-pass metabolism, quick elimination, and poor skin retention. Topically, mild to severe PSO can be treated with glucocorticoids, vitamin D analogs, and phototherapy. Systemic therapy is frequently required for moderate to severe psoriasis. Because the etiology of PSO is widely undefined, and patients develop a variety of symptoms that indicate the illness, ranging from light and small plaques to profound and large plaques with varying characteristics, care for this illness is highly empirical. Unfortunately, there is no known cure for PSO; therapy tries to minimize symptoms and enhance the quality of life [86].

According to the classification of PSO, there are three treatment options: topical, systemic, and phototherapy. Milder topical therapies may be indicated for those with mild to severe PSO. When topical and systemic therapies fail, phototherapy is usually recommended for people with mild to severe PSO.

### 5.1. Topical Therapy

The first defense mechanism is topical therapy, which involves applying the medication to the affected skin. These topical formulations are commonly available in creams, lotions, salves, and shampoo forms. To treat PSO, a variety of topical therapies with various modes of action are available. Topical medications are convenient and easy to use, but they typically work better when combined with other topical treatments. Dithranol (also known as anthralin) is among the ancient therapies for plaque PSO [86], and it works by suppressing proliferation and causing keratinocyte death in HaCaT cells. In fact, the direct or indirect actions of dithranol on the inflammation of PSO are not completely understood, despite numerous studies [87].

### 5.2. Photo-Based Therapy

**Figure 4:**
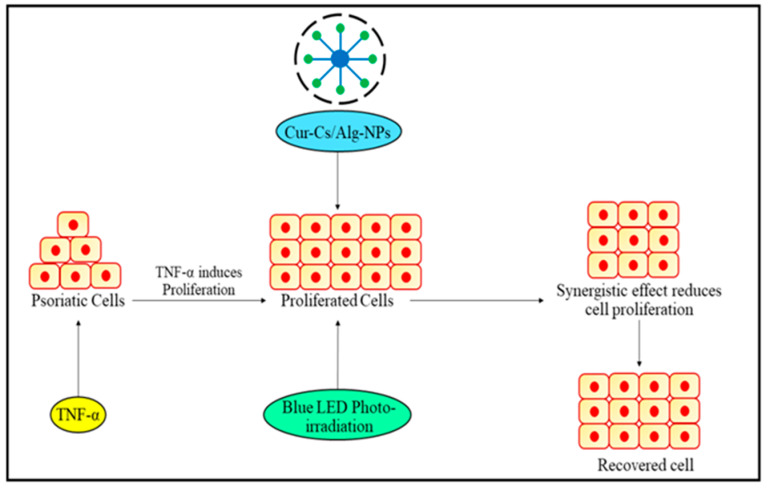
Revitalizing the affected Psoriatic cell; approach using blue LED photo irradiation and nanoparticles for enhanced cell proliferation and recovery.

Photo-based therapy has been used to cure PSO since the 1920s and is now a standard remedy for mild to chronic PSO [88]. PDT is predicated on the introduction of a non-toxic photocatalyst locally or systemically, accompanied by illumination using a specific intensity to produce reactive oxygen species (ROS), primarily lethal protonated oxygen. When contrasted to conventional medications, light technique procedures have comparatively few adverse reactions, including immunodeficiency [89]. Current standards prescribe three forms of radiation treatment interventions to alleviate PSO: narrowband UVB (NB-UVB), excimer laser/lamp (targeted phototherapy), and psoralen plus UVA (PUVA) [90]. Blue light illumination has also been shown to control the growth and development of cell cultures [91]. PDT is a technique that has three aspects: visible light, a photocatalyst, and oxygen [92]. A photocatalyst/photosensitizer is delivered and snapped up by specific receptors in a PDT device, after which a calibrated quantity of visible region of the proper frequency is utilized to incinerate the targeted spot. PDT might suppress cell growth and inflamed reactions based on the amount of photosensitizer and radiation employed in the environment [93,94]. The creation of a PDT technology that is customized to generate the appropriate impact on HaCaT activation could thus be beneficial in the treatment of PSO. As a result, the purpose of this work sought to create a PDT apparatus suited for the management of PSO employing curcumin-loaded chitosan/alginate NPs (Cur-CS/Alg NPs) as photocatalysts and blue emitting diodes (LED) as the radiation output as shown in Figure 4.

### 5.3. Systemic Therapy

Systemic therapy makes use of a medication that circulates throughout the body. It is divided into two categories: (1) oral agents and (2) biological agents that are injected or supplied intravenously. If topical and phototherapy are considered ineffective, systemically nonbiologic and biologic treatments may be explored to achieve skin clearance, according to international PSO treatment recommendations. There are now 11 kinds of FDA-approved biologic medicines for adult PSO therapies, which are categorized according to their cytokines categories (TNF, IL-12, IL-23, IL-17A) [95]. The three most common conventional systemic therapies are MTX, cyclosporine, and retinoids. The primary principles of systemic treatments are to inhibit the immunological response and decrease epidermal cell development [96]. Since the administration of systemic medicines is linked to an enhanced vulnerability to infectious diseases as well as a larger probability of consequences from some liver-attenuated illnesses, immunization might have an impact on avoiding or lowering this threat [97].

## 6. Applications

Topical drug delivery is a field of recent research with great clinical implications. In contrast to the development of targeted systemic treatments and biologics, improved topical drug delivery is focused on the great majority of PSO patients with mild to moderate disease. Various anti-inflammatory drugs and herbal compounds are under investigation. Hopefully, a number of these topical treatments become available for dermatologic practice. J. H. Lee et al. carried out small-scale human research on PSO individuals who were resistant to external calcipotriol/betamethasone emollient medication [98]. Calcipotriol (CPT) serves as the initial topical medication used to treat psoriatic vulgaris. It is a structural analog of calcitriol, a natural form of vitamin D, that may preferentially attach toward the receptor for vitamin D and have a genetic regulatory impact, resulting in a reduction in aberrant proliferation as well as differentiation. CPT also lowers the number of T cells in psoriatic lesions and slows the inflammatory response [99]. With its minimal surface tension, an emulsified version is a preferred formulation for transdermal drug delivery. It is simple to disseminate and moisturise when delivered subcutaneously, altering the skin structure and enhancing medication absorption, making it ideal for the therapy of inflammatory cytokines, PSO, particular erythema, as well as other skin disorders [100]. Exopolysaccharides (EPS) have strong moisturizing qualities. EPS are organic lengthy polymeric materials. Since they can store many times, if not hundreds of times, more water, they are frequently utilized as a moisture barrier. Moreover, EPS has a high emulsifying activity and emulsion stability, which are two of its essential physicochemical qualities. Its spatial stability results in the formation of an extensive network in the continuous phase, which helps to stabilize the emulsion. Over decades, researchers have found several EPS from various strains that have demonstrated considerable emulsifying and stabilizing capabilities of the emulsion at low concentrations (about one percent). In conclusion, EPS has a high potential for use in medicinal products as moisturizing ingredients and emulsifiers. Some EPS has indeed been reported to be used in the treatment of PSO [101].

Some of the significant developments in the field of PSO research include:

1. Development of novel gene loci linked to PSO: Genome-wide association studies (GWAS) have revealed many distinct genomic loci linked to PSO, showing knowledge of the disorder’s etiology.
2. Monitoring technologies that have been upgraded: Imaging tools like optical coherence tomography (OCT) and confocal microscopy have made it easier to identify and track PSO [101].
3. Innovative medicinal strategies: Biologic medicines that address certain immunological system elements are now conventional PSO therapies. Further, new medicines are invented, such as small molecules, genetic treatments, and stem cell therapeutics [102].
4. The skin microbiome’s dysbiosis has been linked to the emergence of psoriasis, according to research. As a result, various studies are concentrating on designing PSO-specific microbiome-based therapeutics.
5. Artificial intelligence and neural networks: There is growing curiosity about employing artificial intelligence and machine learning to create improved psoriatic evaluations and therapy methodologies [103].

## 7. Biosafety

Pharmacological treatments such as cyclosporin, MTX, and apremilast have several disadvantages, including large doses and side effects such as vomiting, anxiety, gastrointestinal distress, and upper respiratory infection. Due to the skin’s natural barrier qualities, conventional topical medicines like ointments, creams, and gels have poor penetration and low absorption. The growth of stiff scales and hyperkeratosis in psoriatic skin further prevent medications from penetrating the skin. As a result, when standard topical dosage forms are applied, there is a low rate of drug absorption. Therefore, to establish clinical efficiency, repeated use of conventional delivery devices at high doses is required. In addition, the persistence of a dose may cause skin irritation and other negative effects. Topical drug delivery technologies have been investigated for the delivery of therapies to get around these restrictions [104,105,106]. The urgent need for novel treatments is also brought by the notion that the scope and application have several negative adverse effects, such as skin shrinkage, susceptibility to sunlight, itchy skin, increased cancer risk, malignancy, inflammatory diseases, and medical complications [107,108,109]. PSO has been connected to several cardiovascular diseases as well as metabolic disorders. Undiagnosed diabetes, hypertension, or high cholesterol affects around 20% of patients with PSO. Moreover, up to 60 percent of patients with PSO have untreated comorbidities, particularly those associated with vascular disease. Inadequate management can lower a patient’s life span by as much as four years [110].

## 8. Future Perspectives and Conclusions

PSO is regarded as a persistent and severe non-communicable disease or illness, and individuals who suffer from it have a very visible and sometimes stigmatizing condition. In order to enhance both oral and conventional psoriasis therapies, nanotechnology provides promising drug delivery system technologies. Considering higher potency, increasing API absorption to the site of action, and minimal toxicity effect, evidence has suggested the use of nanotechnology in subcutaneous systems as a future strategy, as the skin is in constant contact with the outside site and acts as a barrier that is impermeable to exogenous molecules. For this reason, it is necessary to create new therapies for non-toxic and efficaciously delivering medications to the skin by getting past the natural barrier. Also, the determination of PSO prevalence is critical because these statistics educate and express the illness’s epidemiologic burden to doctors, patients, researchers, policymakers, as well as other stakeholders in their attempts to better the lives of individuals with this condition. The rising incidence found in other nations might be attributed, at least partially, to improved awareness about PSO or a longer lifespan. With population increase and aging, as well as the fact that PSO mostly affects adults, the psoriatic burden may continue to climb. PSO is a complicated, complex illness for which new medicines have emerged in recent years. Despite the effectiveness and safety of targeted medicines, wider drugs remain the majority of systemic psoriatic therapy in so many clinical settings across the world due to cost reasons, dosing regimens, and adverse effect characteristics. Growing data suggests that HaCaT not only operates as a trigger at the start of PSO but also as an executioner in a multimolecular network orchestrated by cytokines, which shows the recurrence of PSO. This knowledge and acknowledgment of the relevance of HaCaT in PSO lead to several beneficial medications for PSO therapy. This overview also examines the processes involved in the progression of the disease, as well as the treatment options that have emerged from the dissection of inflammatory psoriatic pathways. The ongoing advancements and medical uses of NPs show promising futures for the provision of healthcare to people.

## Figures and Tables

**Table 1:** Various nano-sized bioactive treatments used in cosmeceutical therapy.

S.No	Bioactive Products	Functions	Reference
1.	Nano anti-aging agents	cause collagen to lose physical appearance	[35]
2.	Nano moisturizers	generate a thin film/moisture-avoiding shield on the body’s external layer	[36]
3.	Nano skin cleansers	encourage the clearance of epidermal layer germs, resulting in less odor formation	[37]
4.	Nano sunscreens	avoid extensive UV ray exposure and discomfort.	[38]

**Table 2:** Various phytoconstituents show characteristics and their functions.

Phytoconstituents	Characteristics	Functions	Reference
Aloe vera	Antiseptic, anti-inflammatory, wound-healing, and anti-diabetic properties.	Enhances the synthesis of collagen and elastin fibers.	[75]
Curcumin	Anti-inflammatory, anticarcinogenic, antioxidant, antimicrobial and wound-healing.	Inhibits the inflammation and that of keratinocyte abnormal proliferation resides on nuclear factor-κB (NF-κB) suppression, TNF-α, IL-1β and IL-6 downregulation.	[76,77]
Nanovitamin C	Enhanced stability and higher antioxidant activity.	Enhances the carrier of the vitamin against apoptotic effects.	[78]
Nanovitamin E	Antiwrinkle, enhanced skin moisturizing, and prevention of skin disease.	Prepared using solid lipid nanoparticle delivery systems with a size equivalent to 292 nm and enhanced skin protection activity.	[79]
Nanoresveratrol	Functional foods for skincare and health.	Enhanced resveratrol bioavailability to the skin to protect against UV radiation.	[80]
Nanogreen tea	Skincare roles such as anti-aging and prevention of UV-induced photoaging.	Techniques enhance the bioavailability of these compounds in cosmeceutical products.	[81]
Nanoquercetin	Efficiency in skin and beauty care through antioxidant activities	Enhanced beauty due to its abundance of OH groups.	[82]
Nanolycopene	Skincare, including anti-aging and antioxidant activity.	Enhanced bioavailability to the skin with potential antioxidant activity. It enhances the penetration of lycopene to the inner cell and nucleus, which could be useful in skin protection and care.	[83,84]

The methodology of plant isolates containing phytoconstituents can be treated utilizing numerous nanoscale processes, such as nanoprecipitation, sonication, and microemulsion procedures, to generate nanostructures for the management of PSO. Plant extracts can be exploited to create novel components for the management of PSO when these substances can be formed, enclosed, or changed to improve phytoconstituent distribution and effectiveness.

## Data Availability

All data available within the manuscript.

## Abbreviations

PSO	Psoriasis
NPs	Nanoparticles
ITNPs	Intrinsically therapeutic nanoparticles
FDA	Food and Drug Administration
API	Active pharmaceutical ingredients
SLNPs	Solid lipid nanoparticles
NLCs	Nanostructured lipid carriers
HaCaT	Human keratinocyte
ROS	Reactive oxygen species
PDT	Photo-based therapy
TEWL	Trans epidermal water loss
NB-UVB	Narrowband UVB
PUVA	Psoralen plus UVA
CPT	Calcipotriol
EPS	Exopolysaccharides
ECM	Extracellular matrix
SC	Stratum corneum
IL	Interleukins
IFN	Interferons
AuNPs	Gold nanoparticles
MTX	Methotrexate
AgNPs	Silver nanoparticles
Cur	Curcumin
CSNPs	Chitosan nanoparticles
NFs	Nano formulations
